# Tree ring segmentation using UNEt TRansformer neural network on stained microsections for quantitative wood anatomy

**DOI:** 10.3389/fpls.2023.1327163

**Published:** 2024-01-08

**Authors:** Miguel García-Hidalgo, Ángel García-Pedrero, Vicente Rozas, Gabriel Sangüesa-Barreda, Ana I. García-Cervigón, Giulia Resente, Martin Wilmking, José Miguel Olano

**Affiliations:** ^1^ iuFOR, EiFAB, Universidad de Valladolid, Soria, Spain; ^2^ Department of Computer Architecture and Technology, Universidad Politécnica de Madrid, Madrid, Spain; ^3^ Center for Biomedical Technology, Universidad Politécnica de Madrid, Madrid, Spain; ^4^ Biodiversity and Conservation Area, Universidad Rey Juan Carlos, Móstoles, Spain; ^5^ Institute of Botany and Landscape Ecology, University Greifswald, Greifswald, Germany; ^6^ Department DISAFA, University of Torino, Torino, Italy

**Keywords:** image segmentation, neural networks, quantitative wood anatomy, tree ring, UNETR, xylem

## Abstract

Forests are critical in the terrestrial carbon cycle, and the knowledge of their response to ongoing climate change will be crucial for determining future carbon fluxes and climate trajectories. In areas with contrasting seasons, trees form discrete annual rings that can be assigned to calendar years, allowing to extract valuable information about how trees respond to the environment. The anatomical structure of wood provides highly-resolved information about the reaction and adaptation of trees to climate. Quantitative wood anatomy helps to retrieve this information by measuring wood at the cellular level using high-resolution images of wood micro-sections. However, whereas large advances have been made in identifying cellular structures, obtaining meaningful cellular information is still hampered by the correct annual tree ring delimitation on the images. This is a time-consuming task that requires experienced operators to manually delimit ring boundaries. Classic methods of automatic segmentation based on pixel values are being replaced by new approaches using neural networks which are capable of distinguishing structures, even when demarcations require a high level of expertise. Although neural networks have been used for tree ring segmentation on macroscopic images of wood, the complexity of cell patterns in stained microsections of broadleaved species requires adaptive models to accurately accomplish this task. We present an automatic tree ring boundary delineation using neural networks on stained cross-sectional microsection images from beech cores. We trained a UNETR, a combined neural network of UNET and the attention mechanisms of Visual Transformers, to automatically segment annual ring boundaries. Its accuracy was evaluated considering discrepancies with manual segmentation and the consequences of disparity for the goals of quantitative wood anatomy analyses. In most cases (91.8%), automatic segmentation matched or improved manual segmentation, and the rate of vessels assignment to annual rings was similar between the two categories, even when manual segmentation was considered better. The application of convolutional neural networks-based models outperforms human operator segmentations when confronting ring boundary delimitation using specific parameters for quantitative wood anatomy analysis. Current advances on segmentation models may reduce the cost of massive and accurate data collection for quantitative wood anatomy.

## 1 Introduction

Forests are critical to terrestrial carbon fluxes and play a crucial role in the future evolution of atmospheric carbon dioxide concentration (Pan et al., 2011; Cabon et al., 2022). Therefore, it is of key importance to understand the responses of trees to environmental conditions for predicting their behavior in future climate scenarios (Seidl et al., 2017). Fortunately, forests can contribute to decipher these questions through the information stored in wood (Olano et al., 2021; Martinez del Castillo et al., 2022). Xylem anatomical adjustments to climate conditions at different time scales (García-Cervigón et al., 2020; Olano et al., 2022) provide basic information to understand the response of trees to climate change and its interaction with intrinsic processes (Piermattei et al., 2020). Tree xylem constitutes a temporal record of individual tree growth and provides a set of characteristics that store information on how environmental factors influenced wood formation. Therefore, studies of time-resolved information stored in annual tree rings have become a powerful tool to predict the impact of global change on terrestrial ecosystems (Wilmking et al., 2020; Sangüesa-Barreda et al., 2021).

The study of tree ring formation using dendrochronology involves a number of techniques to extract the information stored in the wood (Schweingruber, 1996). Recent technical advances in dendrochronological studies have improved data collection and processing using a wide range of tools, ranging from the traditional macroscopic to microscopic perspectives (von Arx et al., 2016; Garcia-Hidalgo et al., 2022). Initially, tree ring studies focused on observing wood samples using a stereoscopic set to determine total ring width and eventually early and latewood measurements, when this annual subdivision could be established, resulting in annually resolved time series of tree growth investment (Schweingruber et al., 2005). Advancements in microsectioning and wood staining along with the recent development of high-resolution digitization tools have facilitated the integration of the microscopic world into the digital realm. This has opened up new avenues of research that concentrate on identifying and quantifying xylem cells at the intraannual level, presenting a plethora of possibilities and research objectives (Fonti et al., 2010; Olano et al., 2013). The term quantitative wood anatomy (QWA) was coined to describe this approach, which gathers comprehensive data on xylem anatomy and provides exciting opportunities to explore intraannual variation and analyze xylem functional properties.

Specifically, QWA provides effective information from measurable features of conductive xylem vessels (e.g., lumen area, cell wall dimension, cell pattern), but also from other wood cells (parenchyma, fibers, resin ducts, etc.), and related properties (e.g., density fluctuations) (Anadon-Rosell et al., 2018; Arzac et al., 2018). By means of these anatomical features, QWA produces precise and valuable measurements, expanding dendrochronology to a deeper knowledge of functional tree traits, such as tree physiology or ecophysiology (Cuny et al., 2012; Pérez-de-Lis et al., 2022). Furthermore, these traits can be transformed into functional features (Hérault et al., 2011; Borghetti et al., 2020). As a time-resolved data, the precise assignment of tree growth to calendar years constrains the use of QWA. However, as secondary tree growth occurs sequentially, annual rings can usually be identified by the cessation of growth when a limiting season occurs. When rings are properly identified and dated, each measured wood feature can be related to extrinsic sources of time-resolved data (*e.g*, climatic features, environmental disturbances, water availability) (Takahashi et al., 2005; Zhang et al., 2021; Anderson-Teixeira et al., 2022).

Anatomical features in wood are currently studied using digital images obtained from wood-stained microsections according to standardized protocols (von Arx et al., 2016; Prislan et al., 2022). Software advances at QWA analyses allow the transition from measuring individual cell traits to semi-automated identification and quantification of large numbers of cells (e.g., 20, 600 xylem vessels in just one core), increasing the validity of the data obtained. Furthermore, some of the available software offers automatic ring boundary detection (Resente et al. *in prep*., von Arx & Carrer, 2014), but there is still ample room for improvement in this task. Currently, most implementations of automatic ring boundary segmentation compute the variation in pixel value through a selected path across the sample image and find the points that exceed an arbitrary threshold. This approach has been widely used for macroscopic wood samples (e.g., increment cores or wood slides) with satisfactory results (Fabijanska et al., 2017; Kim et al., 2023). However, these techniques are less accurate for wood microsections, and in most cases, thorough expert supervision is required. In fact, the low reliability associated with automatic segmentation forces most experts to segment ring boundaries from scratch due to time-consuming amendments.

The recent development of novel hardware (e.g., Graphic Processing Units, GPUs) facilitates the high computational tasks required for image segmentation and high-resolution image analysis, leading to remarkable applications for dendrochronological samples. Out of the wide range of techniques, convolutional neural networks (CNNs) are widely used for image segmentation and achieve remarkable results in different fields (Sultana et al., 2020; Singh et al., 2022). CNNs not only consider the individual pixel value, but also pixel positions and their relationships with the surrounding pixels. Thus, CNNs extract complex information throughout sequential processes (e.g., convolutions, pooling), allowing complex and more abstract information to segment images (Sultana et al., 2020).

CNNs methods accomplish a remarkable ring boundary detection rate for macroscopic samples (Fabijanska & Danek, 2018). Further developments in annual ring recognition and model validation for dendrochronological samples were recently addressed considering ring boundaries as polygons instead of polylines (Poláček et al., 2023) or tree rings as instance segmentations (Gillert et al., 2023). Although these approaches have yielded remarkable results in automated ring boundary segmentation for dendrochronological samples, QWA focuses on cellular and anatomical characteristics of tree rings and requires specific segmentation models and robust methods for model evaluation. Consequently, due to the large differences between diverse kinds of wood, specific models should take into account cell pattern characteristics to achieve high ring segmentation accuracy. Considering QWA purposes, ring boundary evaluation should follow specific criteria according to the image properties at microscopic scales, where discrepancies in segmentation could affect the general methods for segmentation evaluation.

To circumvent these limitations, we suggest investigating the opportunity of supervised learning methods in identifying ring boundaries in microsections of stained wood. However, CNNs have limited ability to capture global relationships between different image regions, as their operations are mainly local and translation-invariant. Recently, Visual Transformers addressed this limitation by introducing the transformer architecture into the visual domain (Khan et al., 2022). These transformers use self-attention mechanisms to capture long-range dependencies between elements in an image, and effectively model the global relationships (Lin et al., 2022).

To fill this gap in QWA, we aim to generate a neural network model (NN) for automatic ring boundary delimitation on stained microsections of wood using attention mechanisms to improve segmentation accuracy, and evaluate whether the validity of NN-based delineations for QWA analyses correspond with manually created delineations. Such an approach would significantly reduce the effort to obtain QWA data.

## 2 Materials and methods

**Figure 1:**
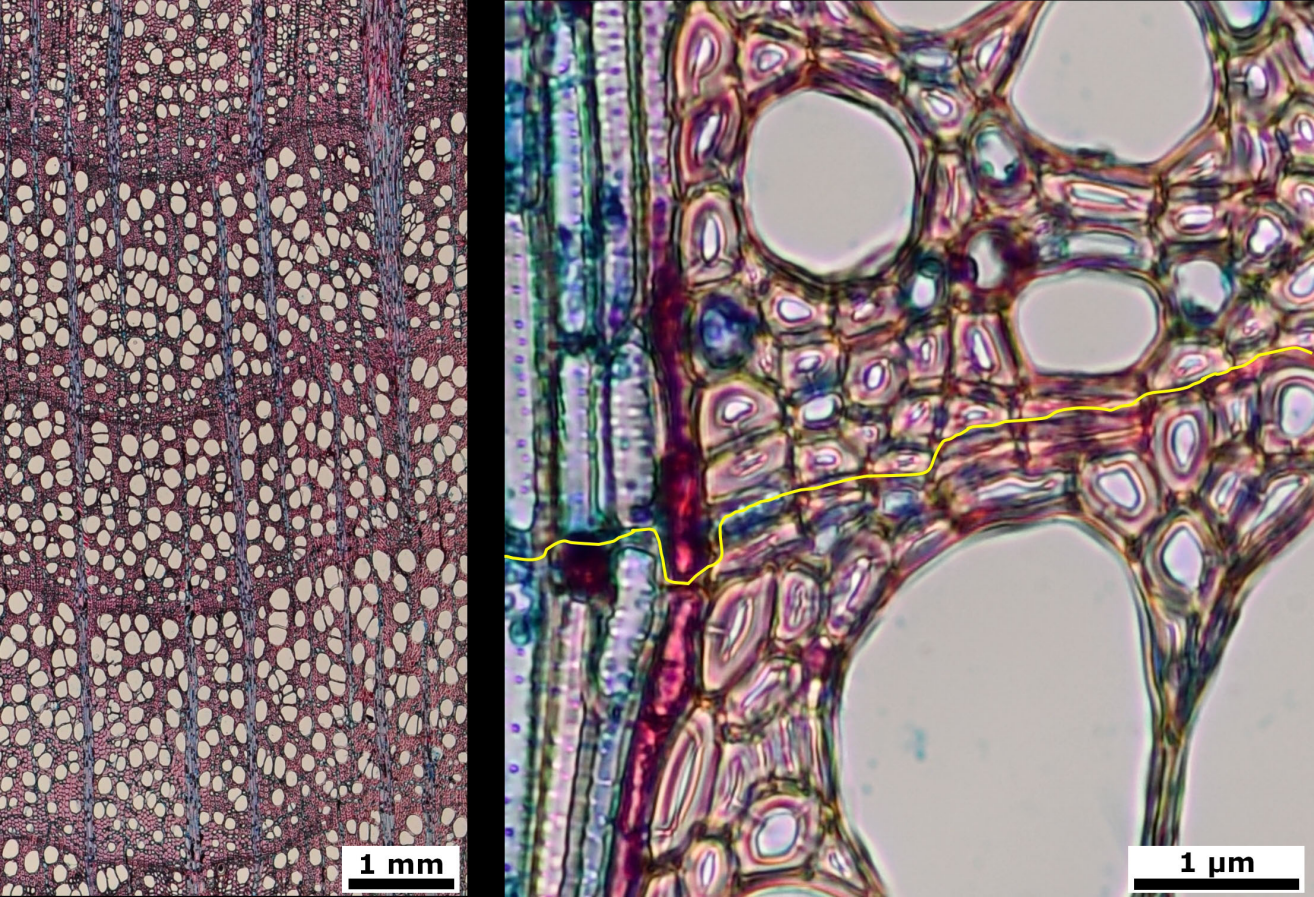
Stained cross microsections from beech (*Fagus sylvatica*) wood cores. 4x image with three full tree rings (left) and 40x detail image with ring limit in yellow (right).

European beech (*Fagus sylvatica* L.) is a deciduous tree species widespread throughout Europe, which represents the broadleaved tree with the highest economic importance for European forestry (Geßler et al., 2007). This species has attracted much interest in dendrochronological studies, including QWA (Sánchez de Dios et al., 2021; Zimmermann et al., 2021) due to the vast distribution range, its economical relevance (forestry) and the dieback that has recently affected this species. Rings usually show a semi-ring-porous vessel distribution i.e. the decreasing lumen diameter in xylem vessels across the annual ring, with distinct rings identifiable by a band of narrower fibers in this species (**Figure 1**).

We developed a UNETR model trained using a collection of 214 transverse microsections of tree cores taken at different climate conditions and elevations derived from previous research (Olano et al., 2022). These microsections were stained with safranin and Alcian blue according to the standard procedures (Garcia-Pedrero et al., 2018) and digitized as RGB images using a Nikon D90 digital camera on a Nikon Eclipse 50i optical microscope at 40x magnification, resulting in a resolution of 1.63 µm per pixel. Individual images were stitched using PTGUI v8.3.10 Pro (New House Internet Services B.V., Rotterdam, The Netherlands).

Two different operators manually delineated ring boundaries on 214 digitized samples using ROXAS software in accordance with the general standards and guidelines for QWA (von Arx et al., 2016).

### 2.1 Data preprocessing

To feed the models, we cropped the images into 256 x 256 pixels, hereafter referred to as data patches. Data augmentation techniques were performed over these patches during training to increase data variability. These procedures included horizontal flipping operations (left-to-right flipping) and rotation with −20° and 20° angles. Furthermore, the patches were randomly blurred with a maximum and a minimum Gaussian kernel size of 3 and 10, respectively. Finally, each data patch was normalized in the range [0, 1]. Data augmentation was performed using the Albumentations library (Buslaev et al., 2020).

**Figure 2:**
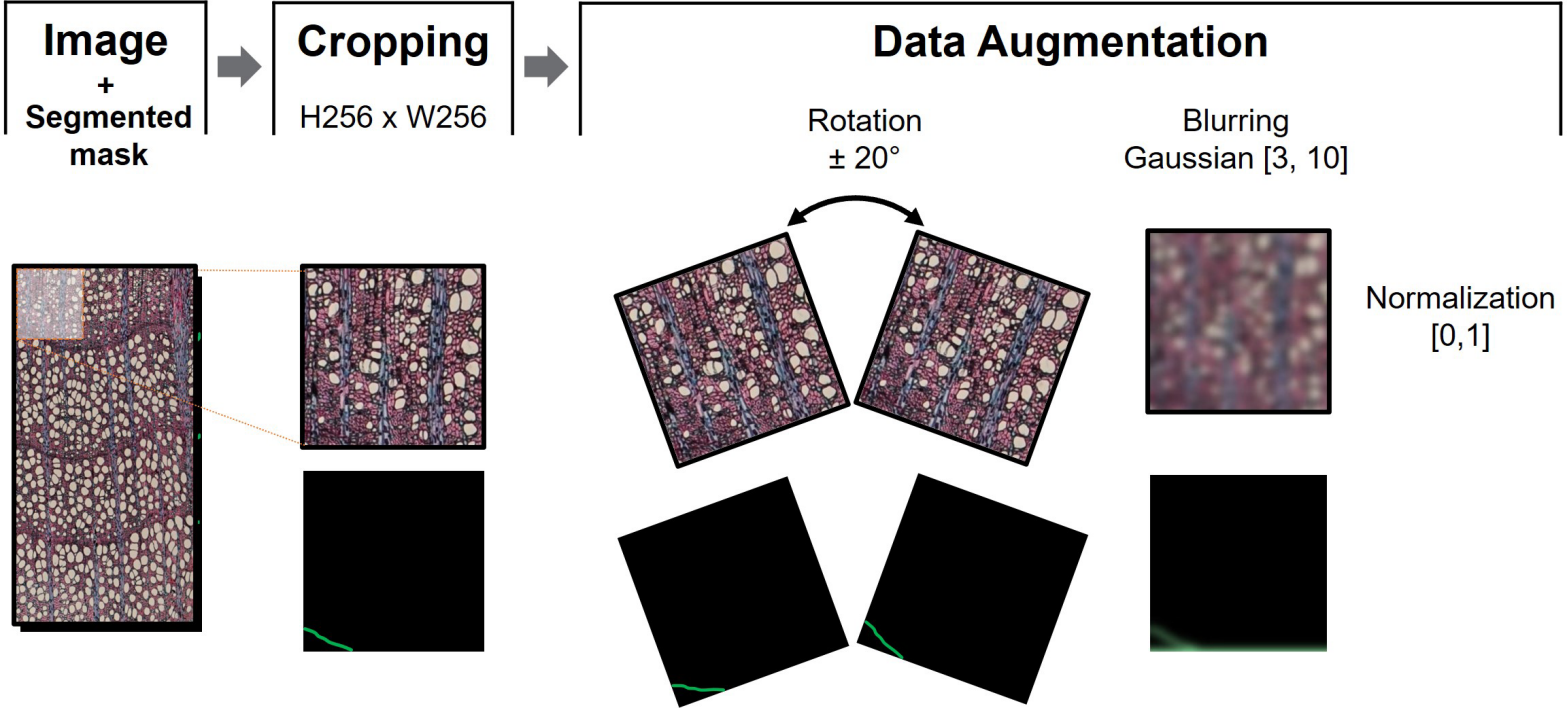
Data preprocessing sequential steps. After cropping the image into N patches of 256x256 pixels, data augmentation consisted of +20° and -20° patch rotation, and blurring using maximum and minimum kernel size of 3 and 10, respectively. The sample image was then normalized in the range [0,1] to train the model associated to the corresponding mask.

Finally, the delimited ring boundaries were binarized to obtain masks where the number 1 corresponded to the pixels belonging to the ring boundary and 0 to the pixels of the background. These masks were subjected to the same processes as the data patches, except for normalization, so that the lines matched the rings. Preprocessing steps are illustrated at **Figure 2**.

### 2.2 NN model

**Figure 3:**
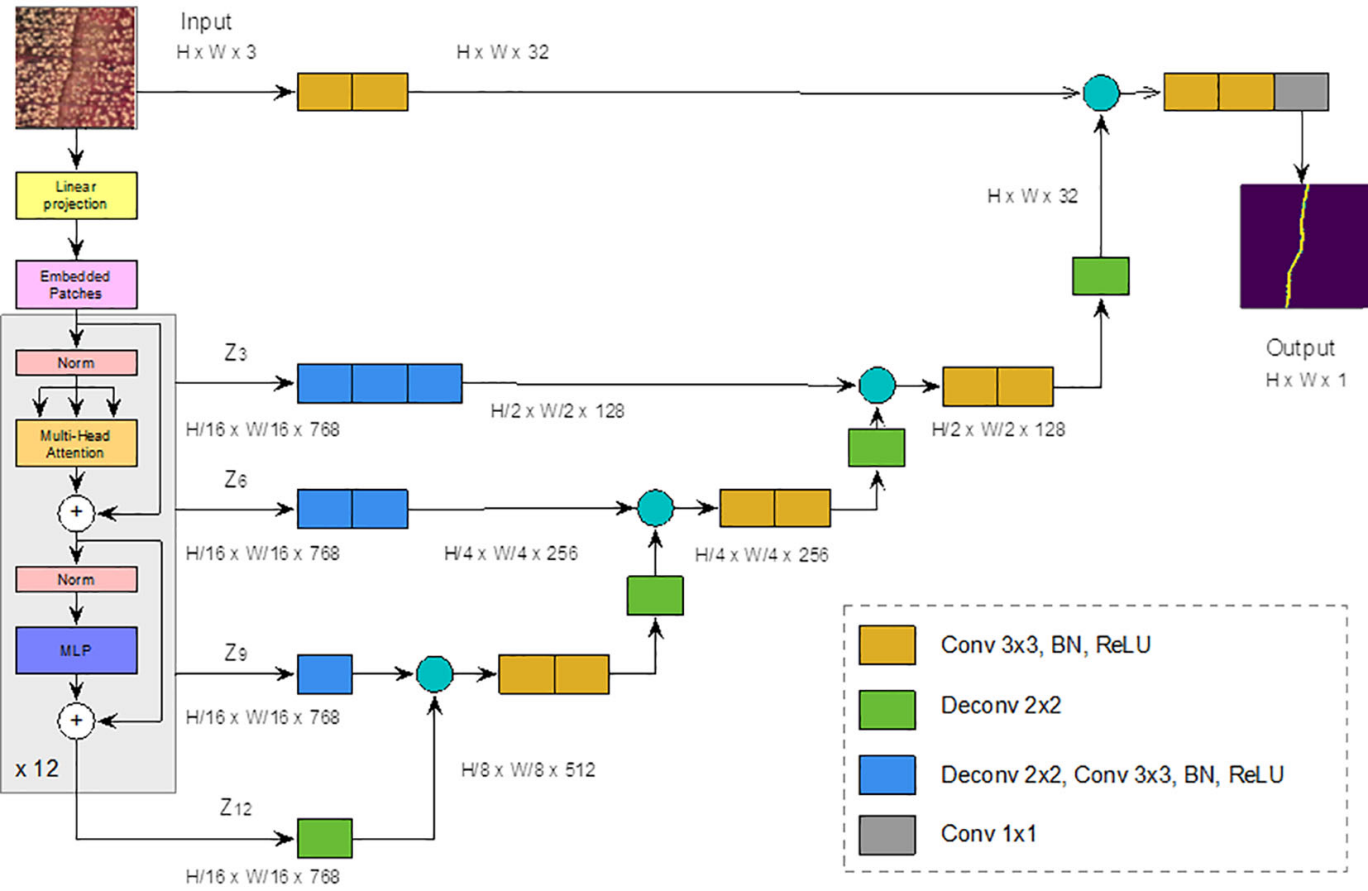
UNETR architecture. Modified from (Ronneberger et al., 2015). The yellow block represents a 3x3 convolution layer, followed by a batch normalization and a ReLU layers. Green Block represents a 2x2 deconvolution layer, blue block represents a 2x2 deconvolution layer, followed by a 3x3 convolution, a batch normalization and a ReLU layers. Finally, the gray block represents a 1x1 convolution.

To distinguish whether a pixel belongs to the ring boundary or to the background, we considered the UNEt TRansformer (UNETR) semantic segmentation model (Hatamizadeh et al., 2022), which is a state-of-the-art neural network for semantic segmentation. Semantic segmentation assigns a class label (i.e., belonging to ring boundary or not) to each pixel and allows the detection of different regions in an image, in our case ring boundaries. UNETR combines the well-known UNET network (Ronneberger et al., 2015) with Visual Transformers (ViT) (preprint Dosovitskiy et al., 2020) (**Figure 3**). ViT outperforms the state-of-the-art CNNs in terms of accuracy and computational efficiency. It uses mechanisms of attention by differentially weighting the importance of each part of the image independently. Considering the small number of pixels belonging to ring border in the complete image of the sample, this self-attention mechanisms should be useful for tree ring boundary. Transformers consist of multiple layers of self-attention. The ViT self-attention layer (Vaswani et al., 2017) allows global embedding of information throughout the image. ViT divides an image into fixed-size patches, linearly embeds each of them, and adds position embedding as input to the Transformer Encoder. In this way, the model learns the training data to encode the relative position of the image patches and to reconstruct the full image structure.

The transform encoder included three layers:

1. Multi-head Self-attention Layer: This layer linearly concatenates all attentional outputs into the appropriate dimensions. The numerous attention heads help to train the local and global dependencies of an image.
2. Multilayer Perceptron Layer (MLP): It contains two layers of neurons with a Gaussian error linear unit (GELU) (preprint by Hendrycks & Gimpel, 2016).
3. Norm Layer: It is added before each block as it contains no new dependencies between the training images. This helps to improve training time and overall performance.

In UNETR (**Figure 3**), input data is divided into a sequence of uniform, nonoverlapping patches and projected into an embedding space, using a linear layer, and projected into an embedding space by means of a linear layer. The sequence is then added with a positional embedding and used as input to a transformer model. The encoded representations of the different layers of the transformer are extracted and merged with a decoder through hopping connections to predict the final segmentation. The output sizes are given for a patch resolution P = 16 (for a total of 256 patches) and an embedding size K = 768.

To train the neural model, we used Focal Tversky Loss (Abraham & Khan, 2019) as the loss function given as follows:


$$ℒ\left(Y,\hat{Y}\right)={\left(1-\frac{\sum_{i}y_{i}\hat{y_{i}}}{\sum_{i}y_{i}\hat{y_{i}}+\alpha \sum_{i}y_{i}\left(1-\hat{y_{i}}\right)+\beta \sum_{i}\left(1-y_{i}\right)\hat{y_{i}}+\epsilon }\right)}^{\gamma }$$

where *y_i_ ∈ Y* and *ŷ_i_ ∈ Ŷ* denote the ground truth and the predicted image of the *i^th^* pixels for the class *C (c ∈* {ring, no ring}) respectively, α and β are the penalty parameters, and ϵ is a smoothing factor used to avoid zero division error. In this work, the following values were used: α = 0.7, β = 0.3, γ = 0.75, and ϵ = 1e−12. The network was trained over 25 epochs, cycles in which the complete training dataset was seen by the network.

### 2.3 Post-processing

**Figure 4:**
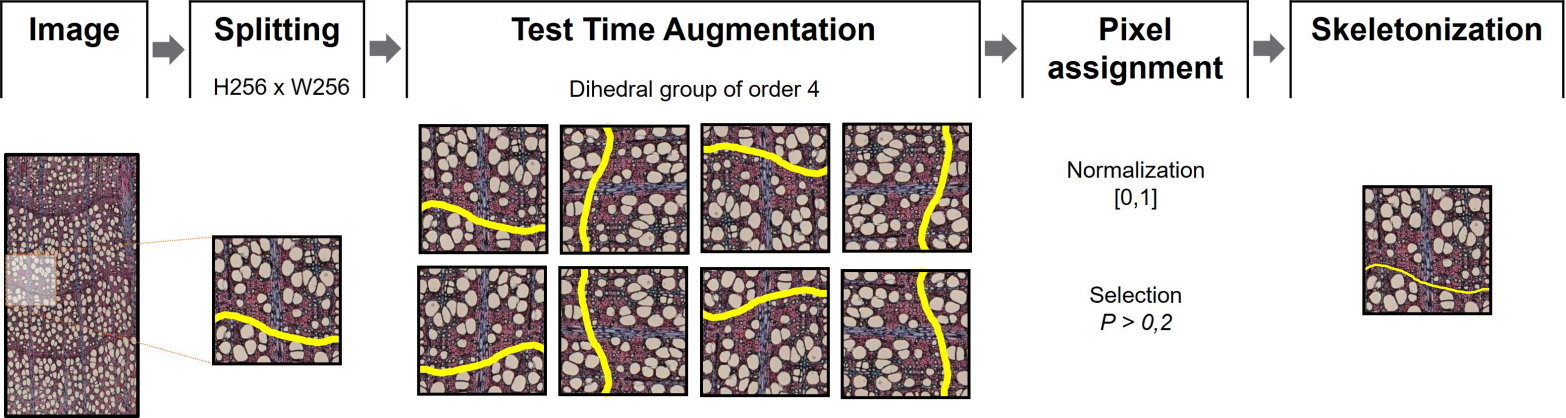
Postprocessing procedures. Images were split into 256x256 pixel patches. Test Time Augmentation (Gonzalo-Martín et al., 2021) was then applied for each patch using an order 4 dihedral group before image normalization [0,1]. Pixels with P > 0.2 on each set of images were considered as boundary. The output segmentation was skeletonized to obtain thin boundaries.

Each high-resolution image was split into N small sub-images, each of the same size than a data patch, **Figure 4**. The sub-images were created using an overlap-tile strategy to an extent of 90% (Ma et al., 2018). To improve the detection of ring boundaries, a test-time-augmentation (TTA) strategy was applied (Gonzalo-Martín et al., 2021). Geometric transformations belonging to the dihedral group of order 4 were used as TTA augmentations, yielding eight different versions of the same sub-image. The probability that a pixel belongs to a ring boundary is found by computing the maximum response of the eight values that correspond to the location of the same pixel. This value is normalized to be in the range [0,1]. A pixel is considered to belong to a ring boundary if it exceeds a threshold of 0.2 (estimated experimentally).

Since prediction networks tend to draw thicker ring boundaries than manually drawn ones, a skeletonization process was performed. This process reduces binary objects to 1-pixel wide representations, providing more appropriate lines for ring boundary delineation (Zhang & Suen, 1984). Because skeletonization generally produces noisy or redundant branches (spurs), a pruning process was performed using the Discrete Skeleton Evolution (DSE) model to preserve only the “trunk” lines with much information (Bai & Latecki, 2007). DSE iteratively eliminates the last branches of the skeleton with less relevance according to their contribution to the shape reconstruction. In this work, branches with an area smaller than 100 pixels were removed. All experiments were performed using an NVIDIA RTX3090 GPU with 10,496 CUDA cores and 24 GB of memory.

### 2.4 Evaluation

To evaluate the results obtained by the segmentation model, a 3-fold cross-validation was performed. In this prospective evaluation, we selected 39 images with both objective and specialist-based analysis of ring boundary delineation. We analyzed 13 images from each fold, including varied parts of the tree trunk from pith to bark and varied lighting.

**Figure 5:**
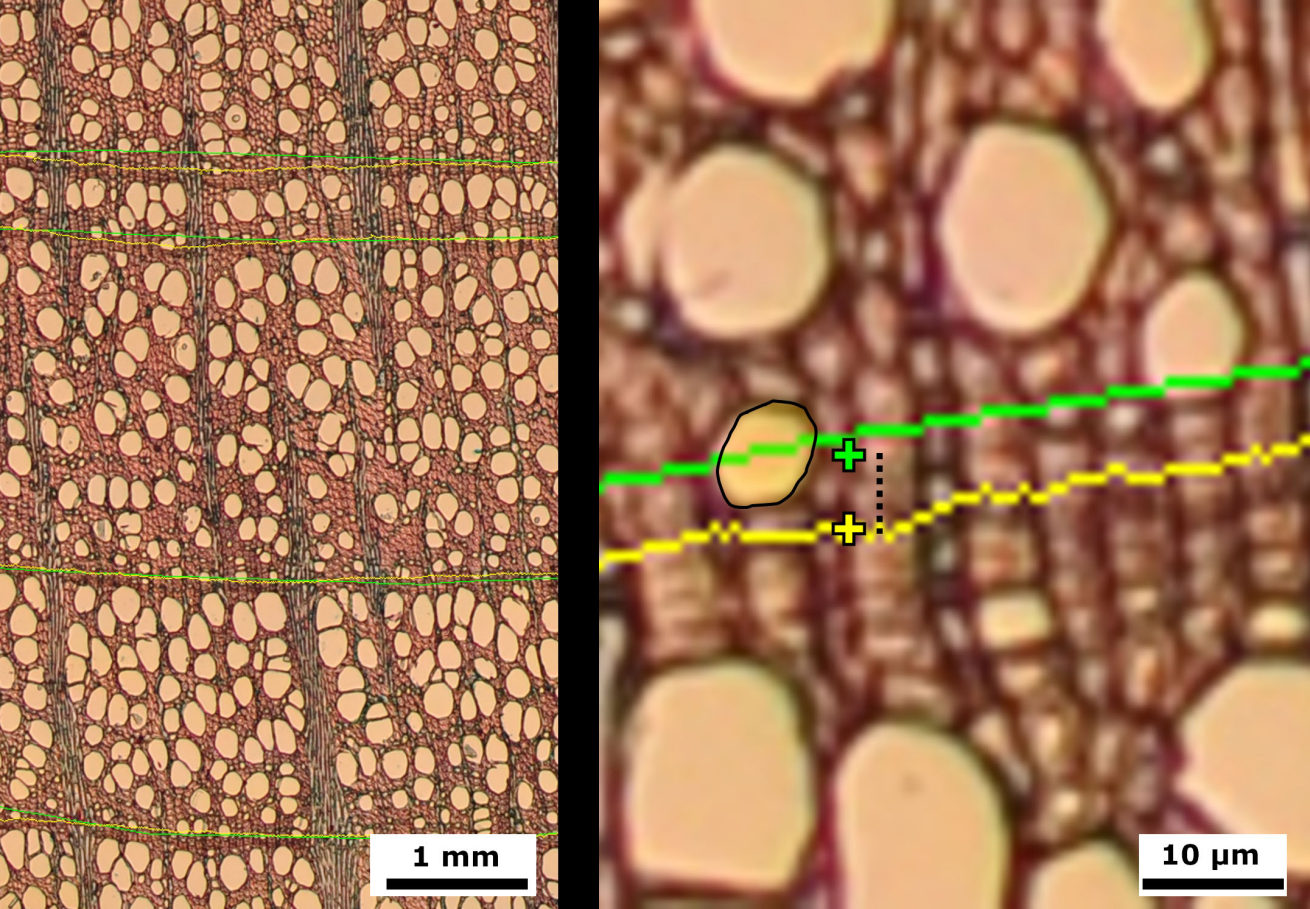
Images of European beech (*Fagus sylvatica*) cross microsections with ring delineations using manual (green) and model (yellow) approaches. Right: Detailed image showing mean ring center (cross), distance between mean ring centers (stripped segment), and vessel with mismatched center (light yellow area with black contour).

Ring boundary are linear paths of 1.63 µm width (1 pixel) delimiting tree rings of 1 110 ± 780 µm width. Thus, pixel by pixel, the discrepancies between the linear paths of ring delimitations severely degrade the results. For example, two complete, equally segmented lines 1 pixel apart result in a 0% match, although the range of error with manual segmentation is commonly larger and practical results for QWA would be equivalent. To enhance the accuracy of the validation method, we compared each ring boundary derived from manually segmented images with those segmented by the NN model according to four criteria (**Figure 5**). (1) We compared pixel coincidence between tree ring boundaries at the pixel level, according to standard image segmentation assessment methods. In a second step, (2) we calculated the mean position of each segmentation for each ring boundary and calculated their spacing. (3) We counted the number of mismatched vessels depending on the ring bordering strategy. Finally, (4) we counted with an expert on QWA to assess which of the ring delimitations performed better according to the ring growth with a trinomial criterion: 0 = better performance of manual delimitation, 1 = no differences, 2 = better performance of NN model delimitation.

## 3 Results

Two different operators manually segmented 5,928 ring boundaries on 214 images used to train and test the model. The test set correspond to 1,329 ring segmentations from 39 randomly selected images, with an image width of 1,468 ± 13.74 pixels (mean ± SE), and a mean height of 23,975 ± 48.61 pixels.

The comparison between segmented boundaries, considering pixel value match, revealed extremely low results (< 1%) as expected. However, the overall mean ring boundary position distance between manual and artificial segmentation was 35.85 ± 3.93 pixels, equivalent to 58.43 ± 6.4 μm. Such a small difference was reflected in the small discrepancy in vessel attribution between delimitation methods, with a mean discrepancy in vessel assignment between models of about a vessel per ring (1.18 ± 0.07 vessels). Expert assessment of the results revealed a solid performance of the NN prediction (**Table 1**), which was better than manual segmentation in 601 (45.22%) cases. In fact, manual segmentation improved the NN in only 108 (8.1%) of the ring delineations. Furthermore, the NN was able to segment 31 borders that were not manually segmented, while failing to detect 16 rings that were segmented by the operators.

**Table 1:** Results of the analysis of discrepancies between segmentations according to expert classification of the best segmentation.

Best ring segmentation (expert decision)	Number of rings (%)	Mean ring center difference (SE) in pixels	Mean vessel mismatch (SE)
**Manual**	108 (8.12%)	34.97 (8.76)	0.97 (0.17)
**Similar**	619 (46.57%)	41.8 (7.02)	0.55 (0.6)
**Model**	601 (45.22%)	21.89 (1.54)	1.9 (0.13)

Discrepancy between the mean ring boundary centers was higher (41.8 ± 7.02) when the segmentations were considered similar by the expert. Meanwhile, ring boundary center difference was 34.97 ± 8.76 pixels when manual delimitation was considered better, and 21.89 ± 1.54 pixels when NN model was judged superior. The largest vessel mismatch between segmentations occurred when the NN model prediction was considered better. In these cases, the trained model included 1.9 ± 0.13 more vessels than manual segmentation. On the other hand, there was less than one vessel mismatch (0.97 ± 0.17) when manual segmentation was considered better.

A separate analysis by folds confirmed the best performance of the NN model segmentation in all the folds (**Table 2**). In fact, the higher performance of manual segmentation was marginal in all the folds, ranging from 6.06% to 9.4% of the segmented ring boundaries.

**Table 2:** Deviations between tree ring boundaries for each fold in the evaluation.

Fold	Best ring segmentation (expert decision)	Number of rings (inner fold %)	Mean ring center difference (SE) in pixels	Mean vessel mismatch (SE)
**1**	Manual	42 (9.4%)	33.56 (3.89)	1.3 (0.33)
	Similar	177 (39.6%)	52.56 (1.51)	0.1 (177)
	Model	226 (50.7%)	25.88 (1.33)	2.13 (0.23)
	Overall	446	37.54 (5.42)	1.36 (0.13)
**2**	Manual	43 (8.53%)	8.78 (1.34)	0.7 (0.22)
	Similar	255 (50.6%)	18.86 (7.45)	0.4 (0.07)
	Model	206 (40.87%)	9.58 (1.35)	1.6 (0.19)
	Overall	504	14.5 (3.92)	0.89(0.09)
**3**	Manual	23 (6.06%)	71.74 (32.82)	1 (0.31)
	Similar	187 (49.34%)	64.15 (17.91)	0.84 (0.12)
	Model	169 (44.59%)	56.93 (13.06)	1.96 (0.25)
	Overall	379	61.41 (10.74)	1.34 (0,13)

## 4 Discussion

The application of NNs in the automation of QWA analyses has been progressive since the development of different cell delimitation models (Garcia-Pedrero et al., 2018, Garcia-Pedrero et al., 2019; Resente et al., 2021). The training and processing effort required of any operator makes it necessary to develop useful methods for ring boundary delimitation on images from microsections. Our model performed equal or better than manual tree ring boundary delimitation on stained wood microsections in 91.79% of the cases.

Beech ring boundaries are characterized by the presence of a band of narrower fibers at the end of the growing season, intersected by parenchyma rays that have less distinct borders. This boundary can be clearly delineated at first glance (at least for the fibers), but conventional manual ring boundary segmentation is not performed pixel by pixel following each cell wall limit, but rather using polylines consisting of segments connecting multiple points marked by the operator. Therefore, the ring boundaries differ slightly between operators, or even between different attempts of a given operator, depending on where the segment limits are placed. This is not a problem with most QWA approaches since most of the effort is based on the assignment of vessels to each ring. Furthermore, these differences are small in terms of percentage of ring or vessel adscription area and subsequently do not affect the robustness or reproducibility of QWA analyses. In this way, our results showed an average spacing of 35.85 (SE = 3.93) pixels with less than one vessel mismatch between manual and automatic delimitations.

The evaluation of a segmentation usually compares ground truth with the predicted boundary at pixel value level (boundary vs. non-boundary). However, this approach has not been widely used in dendrochronological studies, that focus just on tree ring boundary identification. The evaluations usually consider adequate ring boundary detection through visual assessment or partial intersection to identify tree ring boundary (Fabijanska and Danek, 2018). In this way, models achieve remarkable results when applied to macroscopic samples. Higher widening of tree ring border for model evaluation has been proposed as an advance (Poláček et al., 2023) to a finer analysis of segmentations through spatial overlap-based metrics (Taha & Hanbury, 2015).

The particularities of the QWA analysis reinforces the need of a more exhaustive evaluation method. Tree ring boundaries at a single point fix the boundary on a horizontal line, but ring boundaries rarely show this arrangement, resulting in a notable mismatch in the attribution of anatomical structures (vessels, fibers…) to actual rings, *i.e.* calendar years. On the other hand, comparisons at the exact pixel value can lead to a low evaluation of the results when model agreement at pixel level is not exact, even if there are neither vessel nor cell assignment differences. Moreover, a manual ring boundary is usually a polyline linking the different points marked by the operator, whereas the NN model defines boundaries that run through the image pixel by pixel. These contrasting ways of drawing the line, and the serious drawbacks of comparing lines pixel by pixel, requires the use of complementary approaches to evaluating ring boundary detection for QWA. Therefore, we considered the extraction of the mean position of the pixels belonging to each ring boundary as a valuable metric to compare manual and automatic segmentation but complemented with dendrochronological features.

The discrepancies between the predicted and the manually delineated tree ring boundaries were within the expected deviations between different delineations of the same ring, providing a valuable framework for QWA analysis, and allowing the study of tree responses to environmental conditions. The extremely small discrepancy in the number of vessels assigned to a ring (about 1 vessel center discrepancy on average), considering the average of 120 vessels per ring from this data set (Olano et al., 2022), means a very small divergence (<1%). Moreover, the NN was able to identify a significant number of rings that were missed by human experts, while a smaller number were missed.

Using NN-based models in stained images with similar characteristics of wood samples could be useful for broad QWA applications. NNs are sensitive to image properties, primarily resolution and pixel pattern, and require a considerable number of segmented images to learn and modulate the species-specific parameters to achieve optimal results. The growing datasets of wood microsections for QWA open the opportunity for accurate models to segment and, therefore, capture the wide range of cell patterns and wood ring characteristics, including the major anatomical patterns of wood (Wheeler et al., 1989). Furthermore, the differences in tree-ring delimitation between operators and the wide range of wood types highlight the importance of considering not only pixel value but also other relevant metrics for QWA, including expert examination. The compilation of different parameters proposed in this manuscript helps to confront different segmentation methods for QWA.

The proposed UNETR model was developed using beech images following standard microsectioning and data collection protocols. However, there are inherent disparities in image characteristics due to the individualities of trees or even core samples and, similarly, to image capture with a camera on a microscope. Thus, although we followed the standard protocol for QWA samples, NNs can show some degree of bias associated to the training data. In order to apply the proposed model with images different from those obtained for this experiment, we recommend to calibrate the model parameters with a subset of the samples.

Our results show that NNs provide results that have the same or even better accuracy than manual segmentation of ring boundaries, with a slight divergence in vessel mismatch in the marginal cases when NNs models are outperformed by the manual operator. The superior results of NNs can be partially explained by the inherent limitation of manual delineation by polylines, whereas NN can closely match annual tree-ring boundaries to pixel value and pattern. Nonetheless, the great development on the application of NNs at image segmentation in other areas (e.g. biomedical, remote sensing, on-field applications in agroforestry, etc.) reflects that there is still much room for improvement for more specific models according to the wood anatomical patterns, the staining procedures, and the different approaches to model evaluation (i.e., considering the complete ring between two limits as a polygon and examining differences in the segmented area), or even using retraining options with human-corrected results to improve model accuracy. Further advances through this avenue require the compilation of large data banks of images with delineated annual rings on a wide variety of microscopic wood samples.

## 5 Conclusions

CNN models can significantly reduce costs in QWA ring boundary segmentation and have the potential to outperform human operators. Advances in NNs application and the development of specific evaluation methods for wood ring boundary segmentation in QWA could enable the massive collection of accurate data on tree function and response to ongoing global change.

## Data availability statement

The raw data supporting the conclusions of this article will be made available by the authors, without undue reservation.

## Author contributions

MG-H: Conceptualization, Data curation, Formal Analysis, Writing – original draft, Writing – review & editing. ÁG-P: Conceptualization, Formal Analysis, Methodology, Writing – original draft, Writing – review & editing. VR: Conceptualization, Funding acquisition, Project administration, Resources, Writing – original draft, Writing – review & editing. GS-B: Funding acquisition, Writing – original draft, Writing – review & editing. AG-C: Resources, Writing – original draft. GR: Writing – original draft. MW: Writing – original draft, Writing – review & editing. JO: Conceptualization, Funding acquisition, Project administration, Supervision, Writing – original draft, Writing – review & editing.
